# Strengthening Country Readiness for Pandemic-Related Mass Movement: Policy Lessons Learned

**DOI:** 10.3390/ijerph18126377

**Published:** 2021-06-12

**Authors:** Matteo Dembech, Zoltan Katz, Istvan Szilard

**Affiliations:** Medical School, University of Pécs, H-7624 Pécs, Hungary; zoltan.katz@aok.pte.hu (Z.K.); istvan.szilard@aok.pte.hu (I.S.)

**Keywords:** COVID-19, readiness, refugees, migration, health policy, health system strengthening

## Abstract

The COVID-19 pandemic has thus far restricted the large movement of people; nonetheless, we cannot exclude the disruptive power of a virus with similar characteristics to COVID-19 affecting both high- and low-income countries, as a factor for future mass migrations. Indeed, the top 15 countries affected by COVID-19 host about 9 million refugees, and it is, therefore, important to investigate and strengthen the readiness of countries’ health policies to ensure they are well equipped to deal with potential large influxes of ‘epidemic-related refugees and migrants.’ Using the Bardach Policy Framework as a tool for analysis, this article investigates the readiness of countries for a potential public health event (mass migration generated by future pandemics), therefore, aiming at a health response forecasting exercise. The article reviews the policies put in place by countries who faced large influxes of migrants between 2011 and 2015 (the policy-prolific years between the Arab Spring migration and the introduction of stringent measures in Europe) and new evidence generated in response to the COVID-19 pandemic (including the ‘ECDC Guidance on infection prevention and control of COVID-19 in migrant and refugee reception and detention centres in the EU/EEA and the UK’ and the ‘WHO Lancet priority for dealing with migration and COVID-19′) to formulate a policy option able to strengthen national system capacities for responding to influxes of epidemic-related migrants and the management of highly infectious diseases.

## 1. Introduction

The reasons for migration have traditionally been rooted in economic conditions, political unrest, or armed conflict. People leave their homes and livelihoods for a variety of reasons—some flee political instability, war, or human rights violations, while others seek stable work, a more secure living situation, or opportunities which are not available in their home countries [1]. 

The Arab Spring and instability in the Middle East triggered an increasing number of undocumented migrants arriving in Mediterranean countries’ territories and, in particular, affected southern European countries. Affected countries quickly recognized that their health systems were overstretched and not ready to match the health needs of undocumented migrants with existing resources, plans, and policies [2]. European Countries rushed to equip themselves with policies and tools to adequately face this challenge. The period saw a large number of new policies and literature production until approximately 2015, after which the EU witnessed a closure and a slowing down of responsive immigration policies, which culminated in 2016 with the EUNAVFOR MED operation, Sophia, and the EU-Turkey pact, which aimed at closing the frontiers of the EU on the east borders. 

Faced with system unreadiness—and realising the precariousness of their policies, which lacked the capacity for mobilising multiple sectors at the same time, addressing the most vulnerable among migrants, and planning for a surge capacity, countries began to ask for support in revising their policies. 

This situation—weak policies to respond to emergency influxes—is not new and represents a recurring problem for countries in the last decade, reminding us of the necessity to constantly monitor the root causes of mass migration, forecast their consequences, and adopt new public health policies to strengthen the readiness of health systems. At the time of the COVID-19 pandemic, it is, therefore, useful and necessary to consider the potential consequences of a pandemic on mass migration and on existing capacity to respond to health hazards and migration. 

COVID-19 has spread around the world in few months and has challenged countries from social, environmental and economic perspectives. The pandemic has disrupted the global economy and all governments had to react, building on their existing systems, to slow down the transmission of the virus. Nonetheless, COVID-19 has not generated people fleeing high endemic areas in large numbers. One possible reason for this is that the pandemic did not heavily affect countries that usually generate a large number of migrants at first. Moreover, borders were closed in response to the pandemic, which discouraged migration. Nonetheless, the top 15 countries affected by COVID-19 host about 9 million refugees or 3 out of 10 refugees worldwide [3], and we cannot exclude that the disruptive power of a virus with similar characteristics to COVID-19 could be a factor for future mass migrations. Indeed, the pandemic could have unfolded very differently, and it is, therefore, important to investigate and strengthen the readiness of countries’ health policies to ensure they are well equipped to deal with large influxes of ‘irregular epidemic-related migrants’. As was the case during the Arab Spring, even when borders were closed, mass migration occurred.

Research has shown that there is no systematic association between migration and communicable diseases [4]. Any concerns related to the public health impact of mass population movements need to be addressed through well-functioning public health services, by building Migrant Sensitive health Care Systems (MSHCS) ready to protect the health of migrants and the resident community [4]. Excluding migrants’ access to entitlements or access to healthcare in domestic and legal policy frameworks risks increasing transmission, leading to adverse outcomes and inhibiting access to early detection, treatment and negatively affecting public health management [3]. Countries should, therefore, be ready to address the challenges of mass migration movements in the case of a potential scenario where a pandemic starting in a low-income developing country with a similar transmissibility rate of COVID-19 but a higher mortality rate. 

Countries need to recognise the importance of systemic readiness strengthening and prepare for these types of events. As mass influxes resulting from a recent pandemic did not actually take place, the closest academic exercise that can be conducted is to build a model enabling the utilisation of key elements of the pre-COVID-19 migration health policies and new evidence generated during the COVID-19 pandemic to evaluate whether they are adequate to deal with potential future epidemic-related influxes of people. Through this analysis, it is then possible to distil the best migration health policy practices from EU countries and analyse whether existing best practices can inform the new readiness measures that countries need to put in place when preparing for migration influxes and acute epidemics. 

This paper thus reviews the policies put in place by countries who faced large influxes of migrants between 2011 and 2015—the policy-prolific years between the Arab Spring migration and the introduction of stringent measures in Europe—as well as new evidence generated in response to the COVID-19 pandemic to formulate a policy option able to strengthen national system capacities for responding to influxes of epidemic-related migrants and the management of highly infectious diseases. This paper focuses on migrants in irregular situations, including many of the migrants who arrived on European shores and borders, at least until they apply for asylum. 

## 2. Materials and Methods

In order to assess the readiness of current national policies in relation to providing for the health needs of large influxes of migrants, a methodology allowing for the consideration of contextual variables in needed, as migration, and related policies, are intrinsically affected by social and political issues. The Bardach Framework allows for this intersectionality to be considered and is, therefore, used as a framework for analysis in this paper as it provides a methodology that: (a) provides a clear and flexible list of steps for identifying a policy option; (b) recognizes the political nature of health; (c) represents a model that has already been successfully used for the implementation of a health policy. The approach has a clear advantage of allowing for a clear list of steps towards policy design and implementation to be taken with reasonable efficiency and minimum confusion.

The framework has been frequently used to analyse policies for groups with specific health needs, for example, the Bardach Framework, which was adopted in a study on the ‘Charters and choice for students with disabilities in the School District of Philadelphia’ [5] and has also been used to review national guidelines and create a new strategy for improving policies on obesity at a local level [6]. Given this flexibility, the specific needs of migrants and the needs of different sectors can be taken into consideration in the pursuit of an innovative policy solution. The framework allows for the presentation of ‘alternative courses of action that might be taken’ [7], and by comparing alternatives, it is possible to identify whether—with respect to evaluative criteria of interest—one course of action stands out as preferable, and why. Finally, the framework allows for the statement of recommendations, as to which alternative should be selected or whether a new alternative must be designed. Therefore, through this path of examination, it will be possible to rationally identify a new policy option that attempts to address the problem by taking into consideration selected policy outcomes. 

While the step-by-step process of analysis will help to mitigate bias, it cannot be avoided, given that the authors are responsible for selecting the evaluative criteria and related indicators. As the Bardach Framework is often applied by groups of researchers, this is a factor that may introduce bias into the work. 

### 2.1. Evaluation Criteria and the Development of Indicators

As foreseen by the Bardach policy analysis framework, the strengths and weaknesses of the policies identified were assessed according to four criteria and corresponding indicators selected after a literature review of international laws, resolutions, acts, publications and documents issued by International Organizations (EU, UN Agencies, International Agreements). The following four wider documents/guidelines were selected as they are the most relevant to the policy issues under analysis and have the widest support from countries and international organizations:

- Interim Guidance on Scaling-up COVID-19 Outbreak Readiness and Response Operations in Camps and Camp-like Settings (jointly developed by IFRC, IOM, UNHCR and WHO) and ECDC Guidance on infection prevention and control of COVID-19 in migrant and refugee reception and detention centres in the EU/EEA and the UK.
- WHO Lancet priority for dealing with migration and COVID-19.
- World Health Assembly resolution WHA 61.17 on the Health of Migrants, 2008 (WHO, 2008) and ECDC Public health guidance on screening and vaccination for infectious diseases in newly arrived migrants within the EU/EEA.
- IOM Migration Crisis Operational Framework.

The following section outlines each of the four evaluation criteria and conceptualises and simplifies the contents in the form of an objective indicator. The indicator is later used to objectively judge policies and assign them a score for each criterion. 

#### 2.1.1. Interim Guidance on Scaling-Up COVID-19 Outbreak Readiness and Response Operations in Camps and Camp-Like Settings (Jointly Developed by IFRC, IOM, UNHCR and WHO) and ECDC Guidance on Infection Prevention and Control of COVID-19 in Migrant and Refugee Reception and Detention Centres in the EU/EEA and the UK

“The Interim Guidance on Scaling-up COVID-19 Outbreak Readiness and Response Operations in Camps and Camp-like Settings” [8] notes that, as a result of overcrowded conditions without access to basic sanitation, refugees and migrants are potentially at increased risk of contracting diseases—including COVID-19. Inadequate and overcrowded living arrangements present a severe health risk to inhabitants and host populations. The absence of basic amenities, such as clean running water and soap, insufficient medical personnel presence, and poor access to adequate health information are major problems in these settings. Furthermore, refugees typically face administrative, financial, legal, and language barriers to access health systems.

The “ECDC Guidance on infection prevention and control of COVID-19 in migrant and refugee reception and detention centres in the EU/EEA and the UK,” [9] also notes that environmental factors such as overcrowding in reception and detention centres may increase migrants’ and refugees’ exposure to the disease. While there is no evidence to suggest that SARS-CoV-2 transmission is higher amongst migrants and refugees, outbreaks can spread quickly in the absence of adequate prevention measures (such as social distancing, proper hand hygiene, and self-isolation). Therefore, site-specific epidemiological risk assessments must be performed to determine the extent of the risk of COVID-19 introduction and transmission in such settlements, together with case management protocols and the rapid deployment of outbreak response teams if needed. Furthermore, the provision of free and equitable prevention, testing, treatment and care in reception and detention is critical at all times, but particularly in the context of COVID-19.

The guidance further highlights the particular vulnerability of migrants and refugees to the impact of COVID-19 in the wider community. They are over-represented among the homeless population in most member states. The living conditions for homeless refugees and migrants, in which many people live in close contact, make it difficult to follow public health advice, including basic hygiene, quarantine and self-isolation. International migrant workers and refugees can also be affected by “income loss, health-care insecurity, and the ramifications that come with postponement of decisions on their legal status or reduction of employment, legal, and administrative services” [10]. Furthermore, communicating the risks and prevention of COVID-19 with migrant and refugees currently housed in reception and detention centres requires community engagement and health communication strategies that are adapted to meet the different language, cultural and literacy needs of the different populations. There is currently a lack of culturally and linguistically sensitive information available.

The indicator for this evaluation criteria is developed in Table 1. 

#### 2.1.2. WHO Lancet Priority for Dealing with Migration and COVID-19

World Health Organization executives published a comment in *The Lancet Journal* to provide guidance on the effects of the COVID-19 pandemic on refugees and migrants and the need to include them in outbreak response and readiness [10]. The article outlined provisions for countries to scale up readiness for dealing with specific vulnerabilities and barriers in accessing the health services. 

WHO builds on the elements included in the first evaluation criterion described above by including additional consideration for the functions of the health system and of the legal statuses of the population. The guidance highlights that the shortages of medicines and lack of health-care facilities are often observed during health crises and require medium–long term planning. 

WHO also recalled that the legal statuses of migrants can affect their capacity and will to access the health system. This type of barrier, strongly interconnected with the security concerns of a country, can threaten the successful response to any type of outbreak. WHO, therefore, indicates the following actions to its member states:

- Ensure access to safety, health-care services and information.
- Lift all barriers to accessing health services, including language and physical barriers, as well as legal, administrative and financial constraints; avoid forced returns based on fear or suspicion of COVID-19 transmission, and ensure refugees and migrants are not stigmatized, so they are not fearful to seek treatment or disclose symptoms.

The indicator for this evaluation criteria is developed in Table 2. 

#### 2.1.3. The World Health Assembly Resolution (WHA 61.17) on the Health of Migrants and ECDC Public Health Guidance on Screening and Vaccination for Infectious Diseases in Newly Arrived Migrants within the EU/EEA

The WHA resolution lists issues to be addressed by governments, including communicable and non-communicable diseases, health information and respect for health rights [11]. Particular attention is given to the provision of health care services sensitive to the needs of migrants, taking into consideration cultural, religious, linguistic and gender requirements, together with barriers to health care in the country of arrival. 

The main public health goals of the Resolution WHA61.17, operationalised under the ‘Strategy and action plan for refugee and migrant health in the WHO European Region’ [12], which calls upon member states are:

- To promote migrant-sensitive health policies;
- To promote equitable access to health promotion, disease prevention and care for migrants, subject to national laws and practice, without discrimination on the basis of gender, age, religion, nationality or race;
- To establish health information systems in order to assess and analyse trends in migrants’ health, disaggregating health information by relevant categories;
- To devise mechanisms for improving the health of all populations, including migrants, in particular, through identifying and filling gaps in health service delivery;
- To gather, document and share information and best practices for meeting migrants’ health needs in countries of origin or return, transit and destination;
- To raise health service providers’ and professionals’ cultural and gender sensitivity to migrants’ health issues;
- To train health professionals to deal with the health issues associated with population movement;
- To promote bilateral and multilateral cooperation on migrants’ health among countries involved in the whole migratory process;
- To contribute to the reduction in the global deficit of health professionals and its consequences on the sustainability of health systems and the attainment of the millennium development goals.

##### Addressing the Challenge of Communicable Diseases

Concerns about the public health impact of mass population movements are often focused on the spread of communicable diseases. These concerns need to be addressed through well-functioning public health services, including surveillance and health protection; necessary and proportionate interventions; good public and community information. A proper approach will aim to protect the health of migrants and the resident community, eliminating cultural, social, security-related, and economic barriers to healthcare access. This may be accomplished by ensuring that costs, legal consequences, languages, and cultural services are in place for all individuals, regardless of their economic and legal status.

Several challenges may limit access to vaccinations for migrants in Europe, such as the difficulty to provide multiple doses at regular time, provision of linguistic and culturally sensitive information, legal registrations, and economic constrains [13]. Specific actions should be taken to address the social determinants and health inequity of vulnerable sub-groups [14].

The ECDC public-health guidance on screening and vaccination for infectious diseases in newly arrived migrants within the EU/EEA suggested that ‘it is likely to be both effective and cost-effective to screen migrants for active TB, LTBI, HIV, HCV, HBV, strongyloidiasis and schistosomiasis, and that there is a clear benefit to enrolling migrants in vaccination programmes and ensuring catch-up vaccination where needed’ [15]. 

The indicator for this evaluation criteria is developed in Table 3. 

#### 2.1.4. IOM Migration Crisis Operational Framework 

The IOM Migration Crisis Operational Framework [16] was developed at the request of IOM Member States, pursuant to their growing interest in the migration consequences of crisis situations. 

The Operational Framework is based on the understanding that States bear the primary responsibility to protect and assist crisis-affected persons residing their territory in a manner consistent with international humanitarian and human-rights law. 

The document highlights the key elements of the Operational Framework, a flexible tool that has been designed to: (a) improve and systematize IOM’s response to migration crises by bringing together its different sectors of assistance within a pragmatic and evolving approach, while upholding human rights and humanitarian principles and promoting longer-term development goals; (b) help crisis-affected populations, including displaced persons and international migrants stranded in crisis situations in their destination/transit countries, to better access their fundamental rights to protection and assistance through IOM support to states; (c) respond to the often unaddressed migration dimensions of a crisis, by complementing existing humanitarian systems as well as other systems addressing peace and security, and development issues; (d) build on IOM’s partnerships with states, international organizations and other relevant actors in the fields of humanitarian response, migration, peace and security, and development. 

Two specific indications of the Framework are of particular relevance:

- Health support: to provide comprehensive migrant healthcare and prevention services during the crisis and throughout the movement process—at the pre-departure stage, during travel and transit and upon return based on existing health systems and evidence-based needs assessments.
- Psychosocial support: to promote, protect and support the well-being of crisis affected populations, with activities aimed at reducing psychosocial vulnerabilities, promoting community resilience and ownership, and supporting aid that considers psychosocial and cultural diversity issues.

Given the sensitivity often associated with the belief that migrants are vectors of communicable diseases (CDs) and, at the same time, the need to assess eventual public health risk between country of origin and country of arrival, particular attention in the contingency plan should be given to the surveillance and screening of CDs.

In this sense, proper contingency planning should be in place as tools and processes for change—helping to define needs, address potential problems, clarify roles, improve coordination and generate practical action.

The methodology of contingency planning should include:

- Analysing potential emergencies;
- Analysing the potential humanitarian impact and consequences of identified emergencies;
- Establishing clear objectives, strategies, policies, procedures and articulating critical actions that must be taken to respond to an emergency;
- Ensuring that agreements are recorded, and necessary actions are taken in order to enhance preparedness.

The key indicator is the presence of a contingency plan that describes how the local government response would be organized alongside local arrangements, which are the foundation of the response. In order to identify roles and responsibilities, a series of stakeholder meetings and field visits should be held at a local level and should be organized jointly with the local health department. The response should follow the principle of subsidiarity: decisions should be taken at the lowest appropriate level, with coordination at the highest necessary level. The document should ideally include the public health responsibilities concerning surveillance, epidemiology, expert advice and public health, as well as including work with local providers of commissioned care to ensure the continuation and refinement of existing mechanisms and to ensure flexible and appropriate plans are in place to respond to the emergency. 

The indicator for this evaluation criteria is developed in Table 4. 

### 2.2. Selection of the Evidence

The policies of four European Union countries that were most exposed to the phenomenon under study were selected for analysis: Greece, Italy, Malta and Spain. These countries had the highest percentual increase in the amount of emergency migration during a 1-year period (2014 to 2015) of mass influxes to the EU [17] and the policies and strategies adopted by these countries therefore provide a good model for comparison. 

#### 2.2.1. Spain 

##### Specific Prescription for Migrants’ Health Care

Concerning health care, the Spanish Constitution of 1978, the General Health Act 14/1986, and the Act 16/2003 on Cohesion and Quality of the National Health System (NHS), state that all nationals—including foreign citizens—are entitled to general health care regardless of their economic, social and cultural conditions. The NHS is defined as “universal and general and, with particular regard to the economic and social conditions of the citizens who use it and shall tend to be free of charge” [18]. However, in 2012 changes were introduced in Spain, which limit healthcare coverage for the most vulnerable populations. As a response to the economic crisis, austerity measures have been translated into cuts in the social services. The Royal Decree Law 16/2012 on universal, public healthcare defines additional criteria that must be met to access healthcare in Spain, with particular limits on health services for undocumented migrants from non-EU countries. Today, undocumented migrants only have the right to access to:

- Emergency medical care, up to discharge from hospital;
- Maternal medical care;
- Paediatric care.

There is no specific legislation for anonymous access to the health services for undocumented migrants.

Evidence suggests that some subgroups of migrant populations are disproportionately affected by infectious diseases, such as tuberculosis, HIV, and hepatitis B and C. Screening and vaccination programmes may not cover the majority of migrants in countries where many arrive through irregular routes [19]. Spain offers only certain vaccinations, such as those against diphtheria–tetanus–pertussis, poliomyelitis and measles–mumps–rubella but has been also implementing the EU-HEP SCREEN pilot project focusing on community outreach and opportunistic screening in primary care to target migrants, significantly increasing vaccine uptake [15].

##### Sectors Involved in the Management of Migration and Related Health Issues

In Spain, most key governance functions related to preparedness for migrant influxes are addressed through the Spanish Ministry of the Interior, hand in hand with the 17 semi-autonomous regional authorities and the 2 autonomous cities of Ceuta and Melilla [20]. A variety of other institutional stakeholders are also involved, including the Ministry of Health; the Ministry of Labour (and the Instituto Social de la Marina (ISM; Social Marine Institute)); the Ministry of Foreign Affairs and Cooperation; the Ministry of Defence and the Armed Forces; the Ministry of Public Works and Transport (which includes the Coast Guard); the Ministry of Finance and Public Administration (which has competency over Customs); local authorities and actors, including police, emergency response services, civil protection services and NGOs.

The main national police and security forces involved in this process include the Spanish Civil Guard and the National Police [21], which are coordinated by the Ministry of Interior. The Department for Civil Liberties and Immigration is responsible for immigration and asylum policies.

Within the Ministry of Labour, entities with relevant competencies over migration include the General Secretary of Immigration and Emigration, which implements national policy on border control, immigration and emigration; and the Directorate General of Migration, which develops and manages the system for receiving and integrating immigrants, asylum seekers, refugees, stateless persons, and persons eligible for temporary protection and other regimens of secondary protection [22].

The General Regional Directorates of Social Services and Inclusion ascribe to the national immigration strategy, which promotes measures to provide temporary shelter and standardize documentation for the immigrant population. The initial reception in border or coastal areas is managed through migrant centres that provide health services, shelter and basic social orientation, along with legal aid and general information. Foreign migrants have the explicit right to free legal representation in administrative and judicial procedures that could lead to the denial of permission to enter or to deport from Spanish territory. Legal representation is also guaranteed in all asylum procedures, as is the right to an interpreter. 

At the local level, the National Police are responsible for coordinating preparedness and response to large arrivals in Spanish territory, and the Coast Guard and Border Control branches of the Civil Guard also have competencies in controlling undocumented immigration. For example, command and reserve units of the Civil Guard patrol the border in Ceuta and Melilla, and the Coast Guard is responsible for coastal rescue [23].

##### Provisions on Human Rights and Vulnerabilities

Law 4/2000 on the rights, liberties and social integration of foreigners in Spain guarantees that all foreigners, including undocumented migrants, have the right to effective legal representation. The regulation on the application of this law (articles 23 and 58) establishes that migrants attempting to enter through unofficial channels should be taken to the police station, where they should be identified and provided with interpretation and legal services, along with the opportunity to request asylum. These facilities should have printed materials in several languages, and migrants should be assisted by interpreters when needed to understand their rights and receive any necessary health or social care (ICAM, 2005). 

##### Emergency Management and Contingency Planning

There is not a comprehensive contingency plan for the management of large influxes of migrants. What does exist are all-hazards emergency operational plans that the Ministry of Interior keep at their disposal to be employed in extreme cases and to be used only when necessary. 

In response to COVID-19, on March 14, the Spanish government introduced a state of emergency. Additional emergency measures were announced on 20 March relating to migrations and asylum seekers and broadly suspended administrative deadlines for the duration of the pandemic [24]. 

The functioning of the National Reception System of International Protection was also adapted, temporarily suspending the obligation for refugees and asylum seekers to have valid documents to receive aid covering the basic needs; interviews for those who applied for this aid are not conducted over the phone to assess the applicants’ degree of vulnerability and prioritise access to the system for those most in need. Except in cases of extreme vulnerability, all transfers and referrals of beneficiaries of the system to temporary reception places is suspended.

“The health system preparedness scenario in the country includes the integration of health services in the reception (CETIs) and internment (CIEs) of foreign nationals, the support of the NHS (as articulated at a regional level), the role of the Border Health Agency (managed jointly through the central Ministry of Health and Ministry of Finance and Public Administration), and the formal collaboration with non-profit-making organizations like the Red Cross” [25]. 

Within a month of the onset of the COVID-19 pandemic, most immigration detention centres were empty, as the authorities understood that the conditions in these centres relating to sanitation and the inability to socially distance posed too high a risk to detainees, staff and the wider public. The closure was the result of collaboration between local and regional authorities cooperating with civil society organisations, the Ombudsman, detention centre directors, and judges.

#### 2.2.2. Greece 

##### Specific Prescription for Migrant Health Care

In spring 2014, Greece adopted a Greek Immigration Law (4251/2014) “Code for immigration and social integration and other provisions” [26], defining the health procedure upon arrival of undocumented migrants. Upon arrival, a medical examination is conducted, and clinical history recorded, a social worker discusses with each individual his or her social history. Finally, a psychologist is assigned to perform the psychological/vulnerability assessment process. 

Ministerial Decision (2745/2013) which defines the triage procedure and the services that should be provided [27] without specific prescription of intercultural mediation or anonymous access to the health services for undocumented migrants. The decision prescribes migrants that are transferred to the first reception centre where health professionals can further examine them. If the first reception centre is full, migrants are transferred to the nearby detention centre. The maximum stay in the first reception centre, by law, is 25 days. During this period, immigrants may apply for asylum. Once the 25 days period is over, asylum seekers are transferred to pre-departure facilities for up to 18 months, while awaiting their asylum application decision [26]. The Greek Immigration Law (4251/2014) states that ministries cover the cost of health services delivered to Asylum Seekers and irregular migrants awaiting deportation in detention centres, but it does not address the coverage of secondary and tertiary care for undocumented migrants [27]. 

A national regulation supporting vaccination offer to migrants is available [28]. Only certain vaccinations, including those against diphtheria–tetanus–pertussis, poliomyelitis and measles–mumps–rubella, are offered. Data on administered vaccines is available, only aggregated in Greece.

##### Sectors Involved in the Management of Migration and Related Health Issues

The Greek Immigration Law (4251/2014) provides for the establishment of a High-Level Coordination Committee allocating the Ministry of Health the responsibility for the management of issues relevant to health of migrants. At national level, the Ministry of Health is involved in such operational plans via the National Health Operations Center (NAHOC) which bears the responsibility of all issues relevant to health. 

The Hellenic coast guards and the Hellenic police are the main actors during the reception process, with the latter also being responsible for the administrative procedure that must be followed. With regard to health services and health care (including first aid) of migrants upon arrival in the Greek entry points, those responsible are the local health authorities and some NGOs are contracted to take over some services. In case of emergency, individuals are transferred to the local hospitals to receive the required treatment. Once this process is completed, personal data are recorded, and all individuals’ photos and fingerprints are taken. The roles and responsibilities of all actors, providing health and social support in the First Reception Center are formalized by a Ministerial Decision (2745/2013) which defines the triage procedure and the services that should be provided [27]. 

A number of ministries share responsibilities on immigration issues including the Ministry of Foreign Affairs, the Ministry of Interior, the Ministry of Public Order and Citizen Protection, the Ministry of Health, the Ministry of Education and Religion and the Ministry of Labour and Social Insurance [29]. The Ministry of Interior has overall responsibility for immigration issues and while Law 3907/2011 gave the jurisdiction for setting up and operating the new integrated asylum and migration management system to the Ministry of Public Order and Citizens Protection.

##### Provisions on Human Rights and Vulnerabilities

The treatment of specific categories of vulnerable asylum seekers is described in Presidential Decree 220/2007 “Responsible authorities and local self-administration agencies shall provide for the special treatment of vulnerable asylum seekers, such as minors, unaccompanied minors, disabled and elderly people, pregnant women, single parents with minor children and persons who have been subjected to torture, rape or other serious forms of psychological, physical or sexual violence (Article 17); special care should be provided for minors in general and unaccompanied minors in particular: access to rehabilitation services for minors who have been victims of any form of abuse, neglect, exploitation, torture or cruel, inhuman and degrading treatment, or who have suffered from armed conflicts, and ensure that appropriate mental health care is developed and qualified counselling is provided when needed.” [30]. Responsible authorities for accommodation shall also ensure that unaccompanied minors are placed with adult relatives or a foster family, in accommodation centres with special provisions for minors and are protected from trafficking or exploitation. 

##### Emergency Management and Contingency Planning

There exists no comprehensive contingency plan for the management of large influxes of migrants. All-hazards emergency operational plans that remain at the disposal of the Ministry of Interior and National Health Operational Institute (under the Ministry of Health) do exist, and they are employed in extreme cases and are to be used only when necessary.

During COVID-19, the UNHCR moved at-risk asylum seekers due to overcrowding in the island centres into apartments and hotels. The only two sites featuring an initial set-up for quarantine on the Islands was Chios and Lesvos which themselves were not fully functional and safe for persons of concern [31]. Persons of concern were self-organising physical distancing since no physical barriers were available. Reception conditions on the island remained dire, with overcrowding and unhygienic conditions. When reception support ended for some recognised refugees, they spontaneously moved to the mainland where many were left without adequate access to services and shelter. 

At the land border, Evros RIC operated as a closed facility, providing containers used for isolation of new arrivals. However, UNHCR raised concerns about this, as detained individuals had no access to registration before transfer to the RIC. In the North Aegean Region, the clinics operating as screening centres were temporarily halted due to lack of funding for medical personnel. They are now running and funded by the Ministry of Interior, an important aspect of COVID-19 response [31].

#### 2.2.3. Malta

##### Specific Prescription for Migrants’ Health Care

The Immigration Act (Chapter 217) and the Refugee Act (Chapter 420) [32] is the main legal instrument of Maltese legislation on migration. 

Health care is essentially free of charge at the point of delivery for all individuals residing in Malta who are covered by the Maltese social security legislation. According to the Refugees Act of 1 October 2001 [32], asylum seekers are entitled to receive state-funded medical care and services. Subsidiary legislation states that, wherever possible, asylum seekers are expected to contribute to health care costs [33]; however, where this is not possible, health care is free.

A national regulation supporting vaccination offer to migrants is available. Malta offers only certain vaccinations, including those against diphtheria–tetanus–pertussis, poliomyelitis and measles–mumps–rubella. Data on vaccination uptake among migrants is available at national level only in Malta [28]. 

##### Sectors Involved in the Management of Migration and Related Health Issues

Migration and asylum issues are the responsibility of the Ministry for Home Affairs and National Security (MHAS). Irregular immigration and border control are managed by the Police Immigration Department. The Agency for the Welfare of Asylum Seekers (AWAS) is a branch of the MHAS set up in 2009 to provide accommodation and other services to asylum seekers and protected persons. These ministries meet under the auspices of the Prime Minister to discuss coordination during periods of large influxes of migrants. 

These ministries operate to take care of migrants and asylum seekers entering the country illegally are subject to administrative detention. Individuals who have entered the country illegally and without applying for asylum, or whose asylum application have been definitively rejected before the 12th month, may be detained for a maximum of 18 months with a view to returning them to their country of origin. After six months of detention for the purposes of removal, the initial review by the principal immigration officer is also reviewed by the immigration appeals board. Transfer from the detention centre is carried out if there are no reasonable prospects of return. After the maximum detention period of 18 months, migrants must be released, regardless of the status of their application [34]. 

##### Provisions on Human Rights and Vulnerabilities

Maltese policy allows for the fast-track release of vulnerable groups, including families with children, the elderly, unaccompanied minors, pregnant and breastfeeding women, and people suffering from disabilities and/or serious physical or mental illness. However, in high-pressure situations, owing to the sheer number of arrivals, migrants in those categories can be detained for several days in migrant detention centres before their vulnerability is assessed. Migrants granted “vulnerable persons” status or humanitarian protection, as well as asylum seekers who have been detained for 18 months, are moved to open migrant centres. Open centres are government owned and managed directly by the AWAS or subcontracted to nongovernmental organizations (NGOs). 

##### Emergency Management and Contingency Planning

In terms of emergency health management, a number of different contingency plans (for example, a pandemic preparedness plan and the Mater Dei Hospital critical event guide) and drafts are being developed in various areas of the health sector, focusing on different threats. However, an inclusive, all-hazard health emergency preparedness and response plan is missing from Malta’s contingency planning.

In case of an emergency resulting from a sudden influx of migrants that overwhelms the capacity of ordinary services (army, police and health system), the Civil Protection Department would be called in to provide humanitarian and logistical assistance. A number of NGOs can also be called to provide support with specialized personnel. The primary responsibility for the coordination of search and rescue activities lies with the Armed Forces of Malta that operate through the Malta Rescue Coordination Centre. There is not a comprehensive contingency plan for the management of large influxes of migrants. There are all hazard emergency operational plans that the Prime Minister cabinet keep at its disposal based on extreme case scenarios and to be used when necessary. 

The IOM is engaged in providing multilingual information on COVID-19 to migrant communities via websites and social media. They are also developing guidelines containing consolidated information on the benefits available to third country nationals without employment/who may become unemployed [35]. Asylum seekers rescued in the central Mediterranean continue to be transferred to private vessels that are normally used for coastal tourism and are being held there under the pretext of COVID-19 [36]. 

#### 2.2.4. Italy 

##### Specific Prescription for Migrant Health Care

Art. 32 of the Italian Constitution states that Italy protects health as the fundamental right of the individual and the interest of the community. It guarantees free medical treatment to all indigents. 

Undocumented migrants from extra EU countries have access to all urgent medical care and to a wide health coverage through a specific system called “STP Temporarily Present Foreigners” consisting of a short-term but renewable anonymous code that is provided to all undocumented migrants once they access the health services and receive healthcare (TUI, 2002).

In Italy with law No. 189, of 30 July 2002, a new set of rules was approved in the field of immigration and asylum. Asylum seekers have the right to receive a National Health System code and access to all health services provided by the National Health System from the moment in which the application is submitted (TUI, 2002).

Italy offers to migrant children and adolescents all vaccinations included in the National Immunization Plan and also extend the vaccination offer to adults. Vaccinations in accordance with the national schedule in force, depending on age [28]. Adults with uncertain or no vaccination history include polio, measles, mumps, rubella, chickenpox diphtheria, tetanus, pertussis, HBV [15].

##### Sectors Involved in the Management of Migration and Related Health Issues

Primary health care in the migrant centres is provided by associations or cooperative societies, which have won contracts tendered by Ministry of Interior to deliver a basic package of health assistance within the centres. Their terms of reference and the provision of the services are regulated by a convention with the Ministry of Interior.

The Provincial Health Board is responsible for provision of health services and management of health institutions in a given territory of competence. The various boards and their reference structures (hospitals, health centres) and services managed by them constitute the National Health System, which ensures the protection of health and medical assistance to all citizens and foreigners regularly resident without any difference of treatment. 

During influxes of migrants, local medical teams are responsible for the first medical triage at the point of entry. Scopes of the triage are: (i) to identify cases that must be referred to hospitals and (ii) to certify absence of infectious diseases that contraindicate moving of the migrant to community centres. In case of migrants arriving by sea, a very preliminary medical triage is sometimes conducted on board of the rescue boat when medical staff are present on board. 

Interventions that local authorities and third sector organizations implementing in collaboration, resulting in a series of actions to ensure shelter, food, individual and socio-cultural integration support, and also accompanying measures and legal counselling, health, social and linguistic.

The standard services that have to be ensured in migration centre by law are (i) assistance in favour of the person (clothing, bedding, personal hygiene, etc.; health assistance; psychosocial assistance; translation and cultural mediation); (ii) food; (iii) cleaning and hygiene of the environment; (iv) structural and infrastructural maintenance; (v) legal support; (vi) administrative assistance to help asylum seekers and international protected to access to the Italian administrative system integration. 

##### Provisions on Human Rights and Vulnerabilities

A monitoring body ensuring that services are delivered properly has been established through the Praesidium project involving Italian Red Cross, IOM, UNHCR and Save the Children as an attempt to harmonize implementation.

Italian legislation provides that unaccompanied children (up to 18 years old) cannot be deported and must be accommodated in a safe place [37]. 

For asylum, art. 32 of this law and the regulations of its implementation [38], established a new procedure for the examination of applications submitted by asylum seekers who ask for the recognition of refugee status based on the Geneva Convention. The request for refugee status recognition is to be submitted immediately, upon arrival in Italy, to the border police. If, in the place of arrival, there are no border police, the request should be submitted to the competent Provincial Police Authority for the territory. A territorial commission for the recognition of refugee status examines applications to the status of refugees submitted by the police authority. 

##### Emergency Management and Contingency Planning

When an influx of migrants overwhelms the local capacity to respond, a state of emergency is proposed by the regional authorities, approved, and officially declared by the Council of Ministries and the Prime Minister or the competent minister. In the case that a state of emergency is declared, the Department of Civil Protection takes over the responsibility to coordinate the emergency response with chain of command, contingency plans, response roles and responsibility clearly defined. The Sicily region has been established and adopted, by law, a comprehensive contingency plan for the management of large influxes of migrants. The contingency plan describes how the local government response is organized along with the local arrangements clarifying roles and responsibilities of individuals and organizations for an effective response.

UNCHR and INTERSOS have launched a digital capacity initiative to support refugee-led organizations to build capacity during health crises [39]. In response to COVID-19, several measures were adopted, leading to the closure of ports to NGO vessels and the discontinuation of activities to coordinate rescue operations and disembarkation of those in distress [40]. 

## 3. Results

In accordance with the methodology, in this chapter, the four national policies are judged and compared using the indicators previously selected.

Table 5 shows the scoring of each country’s policies, according to criteria and related indicators.

## 4. Discussion

The scoring presented in the section above does not pretend to do justice to individual country situations. Instead, it simply aims at identifying national policies to be taken into consideration when distilling golden standards for future policies. With that in mind, the following subsections discuss in greater detail the results of the Bardach Policy Framework conducted in this paper, upon which the conclusions presented in the final section are based. 

### 4.1. Criterion: Interim Guidance on Scaling-Up COVID-19 Outbreak Readiness and Response Operations in Camps and Camp-Like Settings (Jointly Developed by IFRC, IOM, UNHCR and WHO) and ECDC Guidance on Infection Prevention and Control of COVID-19 in Migrant and Refugee Reception and Detention Centres in the EU/EEA and the UK

As the results show, with regard to overcrowding without access to basic sanitation, tangible progress has been reported in Malta and Italy. While Greece and Spain still reported overcrowding, as in 2020. 

When looking at migrant access to healthcare services, all countries have shown commitments towards a reduction in the systematic barriers. On the contrary, when looking at countries’ progress, related to tailored and accessible health information, they are not yet implemented, only one solid and well-structured initiative by ITNERSOS was identified in Italy.

### 4.2. Criterion: WHO Lancet Priority for Dealing with Migration and COVID-19

When analysing if the countries policies consider the potential shortages of medicines and lack of healthcare facilities Malta and Greece national plans entail provisions for migrant facilities. When addressing income loss and healthcare insecurity, Greece, Malta and Spain have implemented measures, which is also thanks to the support of International Organizations that can provide important inputs for future policymaking.

### 4.3. Criterion: WHA 61.17 on the Health of Migrants and ECDC Guidance

When analysing if the countries policies promote migrant-sensitive health policies by providing undocumented migrants with free of charge access to healthcare services, it is possible to observe that Malta and Italy provide such services, while Spain and Greece do not entirely fulfil the framework. In Malta, the Immigration Act (Chapter 217) and the Refugee Act (Chapter 420) [32] prescribe that health care is essentially free of charge, and in Italy, Art. 32 of the Constitution ensures the same by guaranteeing free medical treatment to all indigents. In Spain, following the economic crisis, austerity measures have led to cuts in social services and only few services are free of charge. These changes were introduced with the Royal Decree Law 16/2012. In Greece, the Immigration Law (4251/2014) defines which ministries cover the cost of health services delivered to asylum seekers and irregular migrants awaiting deportation in detention centres, but it does not address the coverage of secondary and tertiary care for undocumented migrants [26]. 

When analysing if countries’ policies promote migrant-sensitive health policies by providing undocumented migrants with facilitated access to healthcare by providing confidential and anonymous access to services, only in Italy is this policy is implemented. In Spain, Greece and Malta, there are no specific provisions for anonymous access to the health services for undocumented migrants. In Italy, undocumented migrants have access to all urgent medical care, and to a wide health coverage through a specific system called “STP Temporarily Present Foreigners”, consisting of a short-term but renewable anonymous code that is provided to all undocumented migrants once they access health services and receive healthcare [41].

When analysing if the countries’ policies promote migrant-sensitive health policies that provide for undocumented migrants by foreseeing the presence of intercultural mediators, Spain is the only country that, with the General Regional Directorates of Social Services and Inclusion, guarantees the right to an interpreter.

With regard to screening and vaccination for infectious diseases in newly arrived migrants, Italy and Spain offer a wider package that includes adults and addresses healthcare challenges of accessing immunization services. While Greece and Malta’s national legislation also supports vaccination of migrants, only certain vaccinations are offered.

### 4.4. Criterion: IOM Migration Crisis Operational Framework

When analysing if the countries’ policies consider the need for an operational framework to respond to migration crisis by establishing a contingency plan for large influxes of migrants that includes a dedicated health component for communicable diseases it appears that countries do not have a specific contingency plan with the exception of Italy. Countries, in the case of an emergency resulting from a sudden influx of migrants that overwhelms the capacity of ordinary services (army, police and health system), activate the Civil Protection Department to provide humanitarian and logistical assistance. Italy has established and adopted by law a comprehensive contingency plan for the management of large influxes of migrants in the Sicily region. The contingency plan describes how the local government response is organized along with the local arrangements clarifying roles and responsibilities of individuals and organizations for an effective response.

## 5. Conclusions

This paper has implemented the Bardach Framework for migrant health policy analysis. Policy evidence from the four European Countries with the highest percentage of migrant influx has been used to populate the framework. Policies have been judged according to criteria and related indicators. The results of the analysis suggest that each of the countries’ policies have some gold-standard factors to learn from. By combining them, it is possible to formulate an improved policy for dealing with migrants’ health concerns. 

Addressing the underlying factors that favour the transmission of communicable diseases in migrant settlements, such as overcrowding and poor sanitation, is a key action that can be taken by countries and represents the base on which to build country readiness. Conducting regular epidemiological risk assessments and planning for all potential hazards related to mass migrations are key to addressing factors that favour the transmission of communicable diseases. Readiness should pivot on plans capable of forecasting the specific needs of migrant populations during a pandemic, including cultural and linguistic needs, and addressing the challenges brought by the consequences of various legal statuses on accessing health services. In this regard, policy options must take into account the rights of migrants, the spread of communicable diseases, the security aspects of immigration and the interest of the host population. Existing migration health policies should be able to resist the load of influxes during a pandemic.

Measuring the ease of access to healthcare and the dissemination of information concerning health rights to vulnerable migrants appears to be a key element for migrants’ health policies and should be taken into account when planning for potential future epidemic-related influxes. However, while this approach is embedded in the consensus documents that were considered when developing and analysing Indicator 1, some obstacles could be expected and indeed encountered in countries with a politically hostile attitude towards immigration.

With regard to intersectoral work, it seems that, across the four countries, there is a good degree of informal collaboration, but without formally institutionalized bodies and structures in place to meet their goals. Measures to facilitate and organize multisectoral cooperation have been widely studied and applied in some cases. While this exercise involves political will, it is easy applicable if emanating from a Prime Minister’s cabinet.

Another important issue is whether policies consider and respect the rights and specific vulnerabilities to be addressed when dealing with asylum seekers, minors and vulnerable groups. While all the countries fully address the right to asylum and of health access for asylum seekers, there are diverse and fragmented measures in place to address the conditions that pose a burden to the access of healthcare and the health status of woman, minors and elderly persons. International guidelines and frameworks, therefore, represent a source of information to inform enhanced policies.

Mass migration is an unpredictable phenomenon and countries need to be prepared to face different levels of influxes and to respond to their respective needs. When the needs of migrants overwhelm the country’s capacity to respond, it then enters into an emergency situation. In these cases, a thorough contingency plan, considering different scenarios and the interaction of various sectors is essential. Whilst all of the analysed countries have experienced sudden and large influxes of migrants, only one of them had such a contingency plan in place. This suggests that there is an important opportunity for countries to learn from past events in this field, and to learn from one another by studying and analysing the implementation of contingency plans where they are in force. Considering the possibility of potential epidemic-related migrations to Europe provides the opportunity to reflect on previous practice and consider the improvement of migration policies: on the one hand the attention devoted to the phenomenon is stimulating policy improvements; on the other hand, the politicization of the phenomenon often tends to scapegoat migrants for their supposed economic impact on a nation’s finance and, therefore, can lead to politicians reducing the services provided.

With these lessons in mind, it is possible to conclude by synthesizing a policy that attempts to utilize the strongest elements of each of the policies taken into consideration. Building on these gold standard policy elements that emerged from the four sets of indicators used in this study, a future policy option should:

- Recognize that multisectoral synergy has more impact, and prescribe an established multi-stakeholder working group to improve response coordination and involve all key institutional and non-governmental actors that play a role in the management of migration. By recognizing the political nature of health, it would be availed by high level political commitment, possibly from the highest level in the country. Among the terms of reference of the intersectoral multi-ministerial group, a focus should be placed on the special care due to inequities and vulnerable groups.
- Aim to reduce overcrowded conditions and ensue availability of basic amenities, such as clean running water and soap, adequate medical personnel presence and adequate health information.
- Plan for site-specific epidemiological risk assessments to determine the extent of the risk of outbreaks in such settlements, together with case management protocols and rapid deployment of outbreak response teams if needed.
- Consider the human rights of undocumented migrants and their vulnerabilities and include a specific provision for responsible authorities to provide for the special treatment of vulnerable migrants, such as minors, unaccompanied minors, disabled and elderly people, pregnant women, single parents with minor children and persons who have been subjected to torture, rape or other serious forms of psychological, physical or sexual violence. Fast-track release should be provided for these vulnerable groups from closed centres.
- Promote migrant-sensitive health policies by providing intercultural mediation and guaranteeing it as a right in order to eliminate cultural barriers and facilitate the use of health services.
- Address logistical challenges, such as adequate stocking of medicines and appropriate surge capacity of healthcare facilities.
- Ensure access to safety, healthcare services and information by lifting all barriers to accessing health services, including legal, administrative and financial constraints. This can also be achieved by addressing income loss and healthcare insecurity during outbreaks. A migrant-sensitive health policy would prescribe that health care is essentially free of charge, guaranteeing free medical treatment to all indigents including undocumented migrants, defining which ministries cover the cost of health services (primary, secondary and tertiary care) delivered to each category of migrants.
- Regulate the provision of confidential and anonymous access to all urgent medical care and to a wide health coverage through a specific system, such as the so called “STP Temporarily Present Foreigners”, consisting of a short-term but renewable anonymous code that is provided to all undocumented migrants once they accede the health services and receive healthcare.
- Address screening and vaccination for infectious diseases in newly arrived migrants, aiming at addressing all the barriers to accessing the health services. This should be supported by efficient documentation flow, which aims at ensuring the completion of vaccination series as well as migrant-friendly strategies and a migrant sensitive healthcare system that facilitates migrant access to vaccination.
- Consider the human rights of undocumented migrants, based on the states’ laws to grant them the possibility to apply for asylum. An effective policy provides specific provision for asylum seekers and their health rights during processing time of the asylum application.
- Establish a contingency plan for large influxes of migrants that includes health scenarios aimed at dealing with emergencies, resulting from a sudden influx of migrants that overwhelms the capacity of ordinary services and adopting it by law. The plan needs to describe how the local government response is organized along with the local arrangements, clarifying roles and responsibilities of individuals and organizations for an effective response.

The considerations above do not pretend to be a ‘one size fits all’ policy, as this is not possible given the contextual and institutional specificities of each country and their level of preparation with regard to pandemics. While the four countries under analysis do, in fact, have commonalities, by virtue of the fact they are all wealthy Mediterranean countries potentially facing sudden significant immigration from future epidemics, a key limitation of this study is that its findings may not be applicable to most developing countries. In addition—because of economic, cultural, historical, and geopolitical factors—the application of the proposed policy might not apply in a country with a different context for immigration and a separate historical background. As a result, future steps based on this study could focus on analyses devoted to the implementation of the proposed policy for single countries on an individual basis. This would possibly be undertaken with a focus analysis on cost-effectiveness in terms of a ratio where the denominator is associated with health gains. 

## Figures and Tables

**Table 1:** Indicator 1.

Indicator	Scoring	
Does the policy under analysis recognize the greater impact of multisectoral synergy by prescribing measures that address the following?	YES	NO
•The reduction in overcrowded conditions without access to basic sanitation, including implementation of site-specific epidemiological risk assessments.	1	0
•Barriers to accessing health-care services in humanitarian settings is usually compromised.	1	0
•Barriers to accessing to adequate health information, considering culturally and linguistically accessible information	1	0

**Table 2:** Indicator 2.

Indicator	Scoring	
Does the policy under analysis include refugees and migrants in outbreak response and readiness plans by including specific provisions for the following?	YES	NO
•Addressing potential shortages of medicines and lack of healthcare facilities.	1	0
•Addressing income loss, healthcare insecurity and the difficulties linked to the uncertainty in their legal status or to the reduction of employment, which can further affect refugees and migrants.	1	0

**Table 3:** Indicator 3.

Indicator	Scoring	
Does the policy under analysis promote migrant-sensitive health policies by providing undocumented migrants with the following?	YES	NO
•Free of charge access to healthcare services.	1	0
•Simplified access to healthcare by providing confidential and anonymous access to services.	1	0
•The presence of intercultural mediators.	1	0
•Screening and vaccination for infectious diseases in newly arrived migrants.	1	0

**Table 4:** Indicator 4.

Indicator	Scoring	
Does the policy under analysis consider the need for an operational framework to respond to a migration crisis by including the following?	YES	NO
•A contingency plan for large influxes of migrants that includes a dedicated health component for communicable diseases.	1	0

**Table 5:** Comparative matrix.

Indicators	Spain	Greece	Malta	Italy
	Scoring: Yes = 1, No = 0			
**Indicator 1 based on IFRC, IOM, UNHCR and WHO and ECDC Guidance** Does the policy under analysis recognize the greater impact of multisectoral synergy by prescribing measures that address the following?				
•The reduction in overcrowded conditions without access to basic sanitation, including implementation of site-specific epidemiological risk assessments.	0	0	1	1
•Barriers to accessing health-care services in humanitarian settings is usually compromised.	1	1	1	1
•Barriers to accessing to adequate health information, considering culturally and linguistically accessible information.	0	0	0	0
**Score sub-total**	**1**	**1**	**2**	2
**Indicator 2 based on WHO Lancet priority for dealing with migration and COVID-19.** Does the policy under analysis include refugees and migrants in outbreak response and readiness plans by including specific provisions for the following?				
•Addressing potential shortages of medicines and lack of health-care facilities.	0	1	1	0
•Addressing income loss, health-care insecurity and the difficulties linked to the uncertainty in their legal status or to the reduction of employment, can further affect refugees and migrants.	0	1	1	1
**Score sub-total**	**0**	**2**	**2**	1
**Indicator 3 based on WHA 61.17 on the Health of Migrants and ECDC Public health guidance on screening and vaccination for infectious diseases in newly arrived migrants within the EU/EEA** Does the policy under analysis promote migrant-sensitive health policies by providing undocumented migrants with the following?				
•Free of charge access to healthcare services.	0	0	1	1
•Simplified access to healthcare by providing confidential and anonymous access to services.	0	0	0	1
•The presence of intercultural mediators.	1	1	0	0
•Screening and vaccination for infectious diseases in newly arrived migrants.	1	0	0	1
**Score sub-total**	**2**	**1**	**1**	**3**
**Indicator 4 based onIOM Migration Crisis Operational Framework** Does the policy under analysis consider the need for an operational framework to respond to migration crisis by including the following?				
•Contingency plan for large influxes of migrants that includes a dedicated health component for communicable diseases.	0	0	0	1
**Score sub-total**	**0**	**0**	**0**	**1**
**TOTAL SCORE**	**3**	**4**	**5**	**7**

## Data Availability

Not applicable.
